# Mortality associated with timing of admission to and discharge from ICU: a retrospective cohort study

**DOI:** 10.1186/1472-6963-11-321

**Published:** 2011-11-24

**Authors:** Kevin B Laupland, Benoit Misset, Bertrand Souweine, Alexis Tabah, Elie Azoulay, Dany Goldgran-Toledano, Anne-Sylvie Dumenil, Aurélien Vésin, Samir Jamali, Hatem Kallel, Christophe Clec'h, Michael Darmon, Carole Schwebel, Jean-Francois Timsit

**Affiliations:** 1 University of Grenoble 1 (Joseph Fourier) Integrated Research Center U 823 - Albert Bonniot Institute, Rond Point de la Chantourne 38706, La Tronche Cedex, France; 2 Department of Critical Care Medicine, Peter Lougheed Centre and University of Calgary, 3500 26th Street NE, Calgary, Alberta, Canada T1Y 6J4; 3 Polyvalent ICU, Groupe Hospitalier St Joseph, 145 Rue Raymond Losserand 75014, Paris, France; 4 Medical ICU, Gabriel Montpied University Hospital, 58 Rue Montalembert, 63003 Clermont Ferrand Cedex 1, France; 5 Medical ICU, University Hospital St Louis, 1 Avenue Claude Vellefaux, 75010, Paris, France; 6 Polyvalent ICU, Gonesse General Hospital, 25 Rue Bernard Fevrier, 95500, Gonesse, France; 7 Surgical ICU, 157 Rue de la Porte de Trivaux, 92141, Clamart Cedex, France; 8 Polyvalent ICU, Centre Hospitalier Sud Essonne Dourdan-Etampes - Siège, 26 Avenue Charles de Gaulle, 91150, Etampes, France; 9 Intensive care unit, Centre Hospitalier Andrée Rosemon, Av des Flamboyants, BP 6006 97306, Cayenne, France; 10 Medical-Surgical ICU, Avicenne University Hospital, 125 Rue de Stalingrad, 93009, Bobigny Cedex, France; 11 Medical polyvalent ICU, Grenoble University Hospital, BP217, 38043 Grenoble Cedex 9, France; 12 Medical ICU, University Hospital Bellevue, 25 Boulevard Pasteur, 42100, Saint Etienne, France

## Abstract

**Background:**

Although the association between mortality and admission to intensive care units (ICU) in the "after hours" (weekends and nights) has been the topic of extensive investigation, the timing of discharge from ICU and outcome has been less well investigated. The objective of this study was to assess effect of timing of admission to and discharge from ICUs and subsequent risk for death.

**Methods:**

Adults (≥18 years) admitted to French ICUs participating in Outcomerea between January 2006 and November 2010 were included.

**Results:**

Among the 7,380 patients included, 61% (4,481) were male, the median age was 62 (IQR, 49-75) years, and the median SAPS II score was 40 (IQR, 28-56). Admissions to ICU occurred during weekends (Saturday and Sunday) in 1,708 (23%) cases, during the night (18:00-07:59) in 3,855 (52%), and on nights and/or weekends in 4,659 (63%) cases. Among 5,992 survivors to ICU discharge, 903 (15%) were discharged on weekends, 659 (11%) at night, and 1,434 (24%) on nights and/or weekends. After controlling for a number of co-variates using logistic regression analysis, admission during the after hours was not associated with an increased risk for death. However, patients discharged from ICU on nights were at higher adjusted risk (odds ratio, 1.54; 95% confidence interval, 1.12-2.11) for death.

**Conclusions:**

In this study, ICU discharge at night but not admission was associated with a significant increased risk for death. Further studies are needed to examine whether minimizing night time discharges from ICU may improve outcome.

## Background

Patients who suffer acute illness and are admitted during the "after hours" (weekends or nights) may be at higher risk for adverse outcome as compared to patients admitted during weekdays [1]. Cavallazzi *et al *recently conducted a meta-analysis of ten studies conducted in adult ICUs and found that while night time admission was not associated with an increased risk, a small but significant increased risk for death was associated with weekend admission [2]. Since, Kuijsten *et al *reported a relative risk for death associated with admission in the afterhours of 1.059 (95% confidence interval 1.031-1.088) among 149,894 admissions to Dutch ICUs [3]. More recently Kevat *et al *reported on 245,057 admissions to Australian ICUs and found an increased risk for hospital mortality associated with admission during evenings/nights (17% vs. 14%; p < 0.001) and during weekends (20% vs. 14%; p < 0.001) [4]. The existence and determinants of afterhours admission effects therefore remains a topic of controversy.

While admission to ICU in the after hours has been closely scrutinized, less attention has been directed to how the timing of discharge from ICU may influence outcome [5-10]. Patient discharges in the afterhours may be a reflection of limited ICU bed capacity and potentially patients may suffer an increased mortality risk due to premature discharge or limited availability of care in the ward setting in the afterhours [5,7-10]. The objective of this study was to investigate whether an after hours effect on mortality may be present among patients admitted to and discharged from ICU.

## Methods

This study utilized an inception cohort design. All data was obtained using the Outcomerea database [11]. All first admissions among adults (≥18 years) between January 2006 and November 2010 with complete admission and discharge dates and times were included. According to French law, this study did not require individual patient consent, as it involved research on a previously developed and approved database.

### The Outcomerea database

Outcomerea is a prospective observational study that includes detailed clinical and outcome data on patients admitted to participating French ICUs [11]. In some cases participants in the Outcomerea group have enrolled consecutive patients admitted to ICU and in others sampling has been performed where all consecutive admissions during a period of time during the year or all admissions to certain ICU beds are included. Data included in the Outcomerea database has been collected by senior physicians in the participating ICUs. For each patient, the data were entered into an electronic case-report forms using VIGIREA^® ^and RHEA^® ^data-capture software (OUTCOMEREA™, Rosny-sous-Bois, France), and all case-report forms were then entered into the OUTCOMEREA^® ^data warehouse. The data-capture software automatically conducts multiple checks for internal consistency of most of the variables at entry in the database. Queries generated by these checks were resolved with the source ICU before incorporation of the new data into the database. At each participating ICU, data quality was controlled by having a senior physician from another participating ICU check a 2% random sample of the study data. A one-day coding course is organized annually with the study investigators and contrast research organization monitors.

### Study protocol

Once all of the admissions to ICU were identified fulfilling enrollment criteria, the following information were extracted for each patient at presentation: age and sex, admission category (medical, scheduled surgery, or unscheduled surgery). Severity of illness was evaluated at presentation to ICU using the Simplified Acute Physiology Score (SAPS II) and sepsis-related organ failure assessment (SOFA) [12]. Knaus scale definitions were used to record pre-existing chronic organ failures including respiratory, cardiac, hepatic, renal, and immune system failures [13]. The requirement for assisted ventilation, renal replacement therapy, and use of corticosteroids at admission was recorded. The presence of sepsis, severe sepsis, and septic shock was established using standard criteria [14]. The length of stay in ICU and ICU and hospital deaths were recorded. A weekend was *a priori *defined by the period from 00:00 Saturday to 23:59 Sunday, days as 08:00 to 17:59, and nights as 18:00 to 07:59.

### Statistical analysis

Analysis was performed using Stata version 11.2 (Stata Corp, College Station, TX). To avoid the assessment of multiple outcomes for a single patient, only first ICU presentations were analyzed from patients with multiple ICU admissions. Normally or near-normally distributed variables were reported as means ± standard deviations (SD) and non-normally distributed variables as medians with inter-quartile ranges (IQR). Means were compared using the Student t test and medians using the Mann-Whitney U test. Differences in proportions among categorical data were assessed using Fisher's exact test for pair-wise comparisons and the chi^2 ^test for multiple group trend analysis. Where data missing occurred they were not replaced and are reported with reduced n.

Logistic regression models were developed to assess the independent effects of day and time of admission to and discharge from ICU on in-hospital mortality. Factors included in the initial models were admission SAPS II, medical/surgical classification, presence of septic shock, decision to forego life sustaining therapy (DFLST) order, variables found to be significant to the p < 0.1 level in univariate analyses, and weekend/weekday and day/evening admission time were included in the initial models. The discharge SOFA score was also included in the discharge timing model. Backward step-wise variable elimination was then performed to develop the most parsimonious models. Discrimination was assessed using the area under the receiver operator characteristic (ROC) curve and calibration using the Hosmer-Lemeshow goodness of fit test.

## Results

A total of 7,380 adult patients were included. Sixty-one percent (4,481) of the patients were male, the median age was 62 (IQR, 49-75) years, and the median SAPS II score was 40 (IQR, 28-56). Admissions were from the emergency department in 3,625 (49%), inpatient wards in 2,973 (40%), other intensive care areas in 415 (6%), and other/unknown in 367 (5%); the median stay in hospital prior to ICU admission was 0 IQR, 0-2) days. Admissions to ICU occurred during weekends (Saturday and Sunday) in 1,708 (23%) cases, during the night (18:00-07:59) in 3,855 (52%), and on nights and/or weekends in 4,659 (63%) cases. Among 5,992 survivors to ICU discharge, 903 (15%) were discharged on weekends, 659 (11%) at night, and 1,434 (24%) on nights and/or weekends.

**Figure 1:**
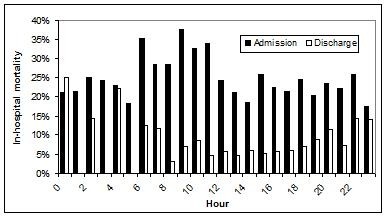
**Mortality associated with hour of admission to and discharge from ICU**.

The overall ICU and in-hospital mortality rates were 1,388/7,380 (19%) and 1,743/7,380 (24%), respectively. The crude risk for in-hospital death associated with time of ICU admission was highest in the late morning as shown in Figure 1. On the other hand, the crude risk for in-hospital death after ICU discharge was lowest during the daytime with rates increasing after early evening and were highest in the early morning (Figure 1). Although admission during night (878/3,855; 23%) as compared to day (865/3,525; 25%); p = 0.079) hours was not associated with mortality, discharge from ICU at night (62/659; 9%) as compared to daytime (293/5,333; 5%; p = 0.0002) hours was associated with subsequent in-hospital mortality.

**Figure 2:**
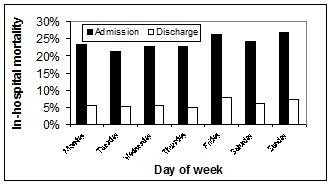
**Mortality associated with day of admission to and discharge from ICU**.

The crude in-hospital mortality rate varied (p = 0.045) according to the day of the week of ICU admission as shown in Figure 2. An increased crude ICU-mortality (353/1708; 21% vs. 1035/5672 (18%); p = 0.026) and overall hospital mortality (432/1708; 25% vs. 1311/5672; 22%; p = 0.005) was observed with admission to ICU during weekends as compared to weekdays. The day of discharge of survivors from ICU was not associated (p = 0.086) with overall risk for in-hospital death (Figure 2). A weekend ICU discharge was not associated with subsequent in-hospital death (60/903; 7% vs. 295/5089; 6%; p = 0.32). Although there was no increased risk for in-hospital death associated with admission during nights and/or weekends as compared to weekday days (1,096/4,659; 24% vs. 647/2,721; 24%; p = 0.82), patients discharged from ICU on nights and/or weekends were more likely to die in-hospital as compared to those discharged during weekday days (111/1,434; 8% vs. 244/4,558; 5%; p = 0.001).

**Figure 3:**
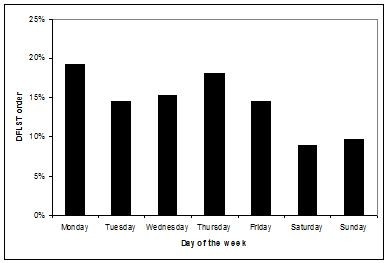
**Day of the week for new decision to forgo life sustaining therapy (DFLST) orders after admission to ICU**.

Patients admitted during weekdays were different based on a number of characteristics from those admitted during nights and/or weekends as shown in Table 1. The overall length of ICU stay was a median of 3 (IQR, 1-7) days and was not different for those admitted on weekends or weekdays (p = 0.075) or in the after hours (p = 0.11). Patients discharged during daytime hours during weekdays were also different from those discharged on weekends and/or nights as shown in Table 2. During the course of the ICU stay, 538 patients had a new decision to forego life sustaining therapy (DFLST) order established. New DFLST orders were less likely to be established on a weekend than a week day as shown in Figure 3.

**Table 1:** Characteristics of adults admitted to ICU during weekday/daytime as compared to nights/weekends

Factor	Monday to Friday (n = 2,721)	18:00-07:59 daily and anytime Saturday or Sunday (n = 4,659)	P-value
Median (IQR) age years	64 (52-76)	62 (48-75)	< 0.0001
Male gender	1073 (61%)	1832/4665 (61%)	0.92
Median (IQR)	39 (27-56)	41 (28-56)	0.0106
SAPS II			
Admit DFLST status	186 (7%)	269 (6%)	0.071
Median (IQR) pre-ICU LOS	1 (0-3)	0 (0-1)	<0.0001
>2 days admit prior	990 (36%)	1101 (24%)	<0.0001
Medical-surgery category			<0.001
Medical	1967 (72%)	3763 (81%)	
Non-scheduled surgery	287 (11%)	585 (13%)	
Scheduled surgery	467 (17%)	311 (7%)	
Admitting surgery			<0.001
Other ICU	153 (6%)	262 (6%)	
Other ward	1364 (50%)	1609 (35%)	
Home	22 (1%)	52 (1%)	
ER	1100 (40%)	2525 (54%)	
Other	82 (3%)	211 (5%)	
Ventilation			<0.001
None	1143 (42%)	2177 (47%)	
Non-invasive	274 (10%)	428 (9%)	
Endotracheal	1304 (48%)	2054 (44%)	
Main diagnostic category			<0.001
Respiratory	662 (24%)	1082 (23%)	
Cardiovascular	375 (14%)	692 (15%)	
Neuromuscular	330 (12%)	694 (15%)	
Gastrointestional	344 (13%)	648 (14%)	
Other surgery	405 (15%)	310 (7%)	
Renal, metabolic,toxic	328 (8%)	761 (16%)	
Infectious	227 (8%)	403 (9%)	
Other	50 (2%)	69 (1%)	
Knaus co-morbidties			
hepatic	176 (6%)	300 (6%)	0.96
cardiovascular	339 (12%)	631 (14%)	0.19
respiratory	363 (13%)	590 (13%)	0.41
renal	173 (6%)	299 (6%)	0.96
immune	409 (15%)	700 (15%)	1.00

Decision to forego life sustaining therapy (DFLST)

**Table 2:** Characteristics of adults discharged alive from ICU during weekday/daytime as compared to nights/weekends

Factor	08:00-17:59 Monday to Friday (n = 4,558)	18:00-07:59 daily and anytime Saturday or Sunday (n = 1,434)	P-value
Median (IQR) age years	64 (52-76)	62 (48-75)	<0.0001
Male gender	2749 (60%)	847 (59%)	0.40
Median admission (IQR) SAPS II	39 (27-56)	41 (28-56)	0.0106
Discharge DFLST status	233 (5%)	61 (4%)	0.21
Discharge SOFA	2 (1-4)	2 (1-4)	0.14
Median (IQR) ICU LOS	3 (1-7)	2 (1-5)	<0.0001
Medical-surgery category			<0.001
Medical	3409 (75%)	1132 (79%)	
Non-scheduled surgery	560 (12%)	167 (12%)	
Scheduled surgery	589 (13%)	135 (9%)	
Main discharge diagnostic category			<0.001
Respiratory	1062 (23%)	291 (20%)	
Cardiovascular	497 (11%)	176 (12%)	
Neuromuscular	611 (13%)	184 (13%)	
Gastrointestional	633 (14%)	206 (14%)	
Other surgery	551 (12%)	129 (9%)	
Renal, metabolic,toxic	716 (16%)	284 (20%)	
Infectious	407 (9%)	133 (9%)	
Other	81 (2%)	31 (2%)	
Knaus comorbidities			
hepatic	279 (6%)	77 (5%)	0.32
cardiovascular	539 (12%)	162 (11%)	0.61
respiratory	569 (12%)	155 (11%)	0.094
renal	279 (6%)	93 (6%)	0.62
immune	638 (14%)	221 (15%)	0.20

Decision to forego life sustaining therapy (DFLST)

Multivariable logistic regression models were developed to assess factors associated with in-hospital death. In the first model (Table 3), neither admission on evenings or weekends to the ICU was associated with increased risk for in-hospital death. In order to assess the potential effect of the timing of ICU discharge on mortality, a second logistic regression model was developed limited to patients surviving to ICU discharge. As shown in Table 4, discharge from ICU during nights was independently associated with subsequent in-hospital mortality. Discharge during Friday, Saturday, and Sundays was associated with an increased risk for death although this was only statistically significant for Fridays (Table 4).

**Table 3:** Logistic regression modeling of factors associated with in-hospital death

Factor	Odds ratio (95% confidence interval)	P-value
Diagnosis renal/toxic/metabolic vs. other	0.30 (0.23-0.38)	<0.001
DFLST order	5.52 (4.28-7.12)	<0.001
SAPS II (per point)	1.07 (1.06-1.07)	<0.001
Male gender	1.31 (1.14-1.50)	<0.001
Pre-ICU hospital stay (per day)	1.01 (1.00-1.02)	0.001
Admission day		
Monday	1 (reference)	-
Tuesday	0.89 (0.69-1.13)	0.337
Wednesday	1.04 (0.81-1.34)	0.737
Thursday	0.99 (0.77-1.27)	0.943
Friday	1.25 (0.98-1.60)	0.078
Saturday	1.07 (0.82-1.38)	0.626
Sunday	1.06 (0.81-1.39)	0.662
Night time admission	0.94 (0.82-1.07)	0.344

The model (n = 7380) had good discrimination (area under receiver operator characteristic curve 0.8543) and calibration (goodness of fit p = 0.411). Decision to forego life sustaining therapy (DFLST)

**Table 4:** Logistic regression modeling of factors associated with in-hospital death following ICU discharge

Factor	Odds ratio (95% confidence interval)	P-value
Male	1.30 (1.02-1.65)	0.031
SAPS II (per point)	1.03 (1.02-1.03)	<0.001
Discharge SOFA score (per point)	1.19 (1.14-1.23)	<0.001
DFLST order	4.55 (3.34-6.20)	<0.001
Diagnosis	1 (reference)	-
Other diagnoses		
Cardiovascular disease	1.50 (1.04-2.16)	0.029
Respiratory disease	1.53 (1.11-2.10)	0.008
Gastrointestinal	1.83 (1.30-2.56)	<0.001
Infection	1.76 (1.20-2.56)	0.003
Discharge day		
Monday	1 (reference)	-
Tuesday	0.99 (0.66-1.48)	0.949
Wednesday	0.91 (0.60-1.36)	0.631
Thursday	0.80 (0.53-1.21)	0.296
Friday	1.45 (1.01-2.10)	0.046
Saturday	1.26 (0.80-2.00)	0.315
Sunday	1.41 (0.84-2.35)	0.193
Night discharge	1.54 (1.12-2.11)	0.008

The model (n = 5,992) had fair discrimination (area under receiver operator characteristic curve 0.771) and calibration (goodness of fit p = 0.4370). Decision to forego life sustaining therapy (DFLST)

## Discussion

In this study we found that while most admissions to ICU occur in the after hours and that weekend admissions were associated with a higher crude case-fatality rate, the day of the week or night time admission was not associated with mortality once adjustment for confounding variables was performed (Table 3). However, the timing of discharge was associated with the subsequent risk for in-hospital death especially with night discharges (Table 4).

Since the initial reports identifying higher neonatal mortality rates associated with weekend deliveries more than 30 years ago there has been hundreds of subsequent publications evaluating potential after hours effects in wide ranges of patients and settings [15,16]. Where after hours effects may be present, it is important issue to define them and explore their determinants. On one hand, an increased risk for death associated with care during different times of the day or week may reflect inconsistencies in availability or quality of care and is a major safety issue that must be addressed. On the other hand, patients admitted in the after hours may be intrinsically at higher risk for death by virtue of a different case-mix, increased severity of illness, or some other unmeasured factor as compared to patients admitted during usual daytime hours.

There is ongoing debate as to whether there may be, and what are the determinants of, after hours admission effects associated with admission to ICUs. In the meta-analysis conducted by Cavallazzi *et al*, weekend but not night time admission was found to be associated with adverse outcome [2]. However, this study has been followed by a large Dutch study that indicated that weekend but not evening admission was associated with adverse outcome [3]. To further the controversy, an Australian study including nearly one quarter million ICU admissions subsequently was published supporting an increased risk for evening/night and weekend admissions [4]. These differences likely at least reflect heterogeneity among study design and different organisational characteristics among participating study ICUs [2]. A number of factors have been suggested to influence after hours effects not limited to reduced nurse and other healthcare worker staffing, closed versus open ICU models, experience and availability of attending intensivists and/or housestaff, and access to treatments and procedures in the after hours [2,3,5-10]. Generally speaking, among our study ICUs while nursing staffing tends to be comparable or only slightly reduced on weekends and evenings, there are significant decreases in allied healthcare workers including physiotherapists and respiratory therapists, and medical staff including residents, fellows, and attending physicians. It is noteworthy, however, that all of our ICUs employed a closed model and that an attending physician is mandated to remain in-house 24/7.

Although much focus has been placed on potential after hours effects associated with the timing of admission, less attention has been paid to course of care subsequent. From the ICU perspective, few studies have evaluated the effects of the time of discharge from ICU and subsequent outcome. While one study from Finland showed no effect [6], others from Denmark [5], The United Kingdom [7], Australia/New Zealand [8,9], and Canada [10,17] found adverse outcome associated with after hours discharges from the ICU. Few clinicians would agree that discharges late at night would be considered to represent optimal care. In most of our participating ICUs weekend day discharges are discouraged and all night discharges are not standard practice. Many weekend and virtually all night discharges are considered premature discharges. These are nearly always due to limited bed capacity in the ICU and need to admit a more acutely or severely ill patient [18]. It is also notable that we observed an increase in risk for subsequent mortality following discharge on Fridays and an increased risk that was not statistically significantly associated with weekend day discharges. We speculate that this increased risk for death could be reflective of decreased intensity of care on wards on weekends. Unlike ICUs which are resource intensive and designed for 24/7 acute care, many other areas of hospitals are typically be less prepared for managing acute patients with consistent support over the hours of night and days of the week. It is also important to recognize that placing limitations on patient care such as restrictions on resuscitation may also influence the outcome of patients after discharge from ICU. We identified that new DFLST orders were less likely to be written on weekends (Figure 3) and that they were important determinants of outcome (Tables 3 and 4).

There are some study limitations that merit discussion. First, it is important to note that there is no general consensus as to what defines "after hours" or weekend care and definitions have varied among studies to date. Our *a priori *selected definitions may not necessarily reflect the exact times when staffing or other service delivery changes may occur, and we did not consider national holidays or seasonal variability. It is notable that a *post hoc *analysis defining a weekend from Friday at 18:00 to Monday at 07:59 and found that this made no appreciable difference in our conclusions. However, in *post hoc *analyses examining six-hourly time periods of 00:00-05:59, 06:00-11:59, 12:00-17:59, and 18:00-23:59 (as suggested by the results in Figure 1), crude in-hospital mortality rates were 22%, 33%, 22%, and 22% (p < 0.001) associated with admission and were 11%, 6%, 5%, and 9% (p = 0.002) associated with ICU discharge during these periods, respectively. Second, this study was a retrospective review. Although data are collected in a prospective manner in Outcomerea, data were not specifically collected for purposes of this study protocol *per se*. While we observed no overall effect on outcome associated with timing of admission to ICU, the possibility exists that certain subgroups of patients, timing of pre-ICU care, or differences in provision of care in the pre-ICU setting may have influenced outcome. A third limitation is that because our study is focussed in the ICU environment, we do not have detailed data on subsequent care provided on wards. As a result we may only speculate as to why patients discharged at night suffered higher mortality. Finally, although multiple centres were included in this study, more than one-half of the patients were enrolled from two ICUs with small numbers included from some sites, limiting the ability to assess inter-facility variability.

## Conclusions

In this study we found no association between the timing of admission to ICU and subsequent outcome after controlling for a number of variables in multivariable analysis. However, the timing of discharge, especially during the night was associated with adverse mortality outcome. Further investigation is needed to examine whether minimization of after hours discharges and/or augmentation of ward care post-ICU discharge may improve the ultimate outcome of critical illness.

## Competing interests

The authors declare that they have no competing interests.

## Authors' contributions

KL and JFT conceived the study and KL and AV performed the data extraction and the statistical analyses. KL wrote the draft version of the manuscript and BM, BS, AT, EA, DGT, ASD, SJ, HK, CC, MD, JFT are clinical investigators of the Outcomerea study groups and critically revised the draft manuscript. All the authors approved the final submission.

## Pre-publication history

The pre-publication history for this paper can be accessed here:

http://www.biomedcentral.com/1472-6963/11/321/prepub

## References

[B1] Bell CM Redelmeier DA Mortality among patients admitted to hospitals on weekends as compared with weekdays N Engl J Med 2001 345 9 663 668 10.1056/NEJMsa003376 11547721

[B2] Cavallazzi R Marik PE Hirani A Pachinburavan M Vasu TS Leiby BE Association between time of admission to the ICU and mortality: a systematic review and metaanalysis Chest 2010 138 1 68 75 20418364 10.1378/chest.09-3018

[B3] Kuijsten HA Brinkman S Meynaar IA Spronk PE van der Spoel JI Bosman RJ de Keizer NF Abu-Hanna A de Lange DW Hospital mortality is associated with ICU admission time Intensive Care Med 2010 36 10 1765 1771 10.1007/s00134-010-1918-1 20549184 PMC2940016

[B4] Kevat DA Davies AR Cameron PA Rajaratnam SM Increased mortality associated with after-hours and weekend admission to the intensive care unit: a retrospective analysis Med J Aust 2011 194 11 616 21644886 10.5694/j.1326-5377.2011.tb03126.x

[B5] Obel N Schierbeck J Pedersen L Storgaard M Pedersen C Sorensen HT Hansen B Mortality after discharge from the intensive care unit during the early weekend period: a population-based cohort study in Denmark Acta Anaesthesiol Scand 2007 51 9 1225 1230 10.1111/j.1399-6576.2007.01431.x 17850563

[B6] Uusaro A Kari A Ruokonen E The effects of ICU admission and discharge times on mortality in Finland Intensive Care Med 2003 29 12 2144 2148 10.1007/s00134-003-2035-1 14600808

[B7] Goldfrad C Rowan K Consequences of discharges from intensive care at night Lancet 2000 355 9210 1138 1142 10.1016/S0140-6736(00)02062-6 10791376

[B8] Duke GJ Green JV Briedis JH Night-shift discharge from intensive care unit increases the mortality-risk of ICU survivors Anaesth Intensive Care 2004 32 5 697 701 15535498 10.1177/0310057X0403200517

[B9] Pilcher DV Duke GJ George C Bailey MJ Hart G After-hours discharge from intensive care increases the risk of readmission and death Anaesth Intensive Care 2007 35 4 477 485 18020063 10.1177/0310057X0703500403

[B10] Laupland KB Shahpori R Kirkpatrick AW Stelfox HT Hospital mortality among adults admitted to and discharged from intensive care on weekends and evenings J Crit Care 2008 23 3 317 324 10.1016/j.jcrc.2007.09.001 18725035

[B11] Outcomerea. Promotion et Development de la Recherche et de l'Enseignement en Reanimation (French) http://www.outcomerea.org. Accessed January 31, 2011

[B12] Le Gall JR Lemeshow S Saulnier F A new Simplified Acute Physiology Score (SAPS II) based on a European/North American multicenter study JAMA 1993 270 24 2957 2963 10.1001/jama.270.24.2957 8254858

[B13] Knaus WA Draper EA Wagner DP Zimmerman JE APACHE II: a severity of disease classification system Crit Care Med 1985 13 10 818 829 10.1097/00003246-198510000-00009 3928249

[B14] American College of Chest Physicians/Society of Critical Care Medicine Consensus Conference: definitions for sepsis and organ failure and guidelines for the use of innovative therapies in sepsis Crit Care Med 1992 20 6 864 874 10.1097/00003246-199206000-00025 1597042

[B15] MacFarlane A Variations in number of births and perinatal mortality by day of week in England and Wales Br Med J 1978 2 6153 1670 1673 10.1136/bmj.2.6153.1670 737435 PMC1609011

[B16] Mangold WD Neonatal mortality by the day of the week in the 1974-75 Arkansas live birth cohort Am J Public Health 1981 71 6 601 605 10.2105/AJPH.71.6.601 7235098 PMC1619835

[B17] Priestap FA Martin CM Impact of intensive care unit discharge time on patient outcome Crit Care Med 2006 34 12 2946 2951 17075364 10.1097/01.CCM.0000247721.97008.6F

[B18] Garrouste-Orgeas M Montuclard L Timsit JF Reignier J Desmettre T Karoubi P Moreau D Montesino L Duguet A Boussat S Predictors of intensive care unit refusal in French intensive care units: a multiple-center study Crit Care Med 2005 33 4 750 755 10.1097/01.CCM.0000157752.26180.F1 15818100

